# Efficacy and safety of efgartigimod in the treatment of impending myasthenic crisis

**DOI:** 10.3389/fimmu.2026.1760953

**Published:** 2026-03-12

**Authors:** Ning Li, Yinghui Zhang, Lu Liu, Yanzi Jin, Li Zhao, Shuhui Zhu, Yating Li, Boya Ma, Jiahui Peng, Juan Yang, Qian Li, Xiao Yang

**Affiliations:** 1 Department of Neurology, General Hospital of Ningxia Medical University, Yinchuan, China; 2 First Clinical Medical College, Ningxia Medical University, Yinchuan, China; 3 Department of Neuroscience Intensive Care Unit (NCU), General Hospital of Ningxia Medical University, Yinchuan, China

**Keywords:** crisis, efgartigimod, FcRn antagonist, immunoglobulin, impending, intravenous, myasthenic, prospective cohort study

## Abstract

**Objective:**

To compare the short-term efficacy and safety of efgartigimod versus intravenous immunoglobulin (IVIg) in patients with impending myasthenic crisis (IMC).

**Methods:**

This single-center, randomized, open-label, prospective comparative cohort study included 38 acetylcholine receptor antibody–positive (AChR−Ab+) patients with IMC, which was defined as rapid progression of bulbar or respiratory symptoms within ≤2 weeks, meeting at least one of the following criteria:Myasthenia Gravis Foundation of America (MGFA) grade IVb, a Quantitative Myasthenia Gravis (QMG) bulbar subscore of 3, a respiratory subscore of 2, or a combined bulbar–respiratory subscore ≥4. The participants were included and categorized into either the efgartigimod group (n=21; 10 mg/kg weekly for 4 weeks) or the IVIg group (n=17; 400 mg/kg/day for 5 days). The primary endpoints were the rate of IMC remission within one month (defined as a QMG score <2 for both the bulbar and respiratory items, or a combined bulbar plus respiratory QMG score <4, or an improvement of the MGFA classification to below class IVb, sustained for at least 24 hours.) and time to remission. Secondary outcomes comprised changes in QMG total, bulbar, and respiratory subscores, and Myasthenia Gravis Activities of Daily Living (MG-ADL) from baseline to remission. Safety was assessed by rates of progression to myasthenic crisis (MC) and treatment-emergent adverse events (TEAEs).

**Results:**

Between January 2024 and March 2025, 38 patients were randomized. IMC remission rates were 90.48% with efgartigimod and 94.12% with IVIg (P = 1.000). The median time to remission was significantly shorter with efgartigimod (8 days; 95% CI: 5.75–10.67) than with IVIg (12 days; 95% CI: 9.92–13.20; P = 0.009). One patient per group progressed to myasthenic crisis (4.76% vs 5.88%). No TEAEs were reported.

**Conclusion:**

Efgartigimod demonstrated comparable efficacy to IVIg for IMC remission but with a significantly faster onset, supporting its role as a rapid and safe alternative.

## Introduction

Myasthenia gravis (MG) is an autoimmune disorder characterized by autoantibodies targeting postsynaptic proteins at the neuromuscular junction, most commonly the acetylcholine receptor (AChR) or muscle-specific kinase (MuSK). Clinically, it presents with fluctuating skeletal muscle weakness and fatigue (1). In some patients, rapid deterioration can culminate in myasthenic crisis (MC), a condition requiring mechanical ventilation and representing a leading cause of mortality in MG (2).

In 2016, the Myasthenia Gravis Foundation of America (MGFA) introduced the term “impending myasthenic crisis” (IMC) to identify patients experiencing rapid clinical decline with a high risk of progression to MC (3). According to the 2024 Chinese MG Collaborative Group Expert Consensus on Management of Impending Myasthenic Crisis (IMC) is defined as significant worsening of bulbar or respiratory muscle function within a short period (≤2 weeks), meeting any of the following criteria: MGFA type IVb; a Quantitative Myasthenia Gravis (QMG) bulbar subscore ≥3; a respiratory subscore ≥2; or a combined bulbar-respiratory subscore ≥4 (4). Given the high risk of progression to MC, the narrow therapeutic window, and the potential for fatal outcomes, timely and effective intervention is critical to reverse functional decline and avert crisis (5).

Currently, intravenous immunoglobulin (IVIg) and plasma exchange (PLEX) are the standard rapid interventions for IMC (6, 7). However, these modalities rely on non-targeted mechanisms, with onset of effect often delayed by several days and benefits lasting only weeks. IVIg also carries risks of thromboembolic events and acute kidney injury and depends on blood product availability, whereas PLEX entails risks of catheter-related infections and potential hemodynamic instability (8). Accordingly, faster-acting, more durable therapies that do not rely on blood products are needed to prevent progression from IMC to MC.

Efgartigimod, an engineered human IgG1 Fc fragment that antagonizes the neonatal Fc receptor (FcRn), accelerates IgG catabolism and reduces circulating pathogenic IgG (9, 10). In the phase 3 ADAPT trial, it demonstrated rapid and sustained clinical benefit with a favorable safety profile in generalized myasthenia gravis (gMG) (11). Observational evidence from case reports, case series, and small retrospective cohorts has also reported rapid improvement in patients with IMC or MC after efgartigimod, supporting its potential utility in acute exacerbations (12–15). Nevertheless, robust evidence in IMC remains limited: no head-to-head randomized controlled trials have compared efgartigimod with standard of care (IVIg or PLEX) in this high-risk population. Its effect size, time to response, and comparative advantages in reversing deterioration, preventing progression to MC, shortening crisis duration, and facilitating recovery require confirmation in adequately powered prospective trials.

To address these evidence gaps, we designed and conducted a single-center, prospective, randomized, open-label, parallel-group clinical trial to directly compare the efficacy and safety of efgartigimod versus IVIg in patients meeting strict diagnostic criteria for IMC.

## Material and methods

### Participants and study design

This prospective, single-center cohort study consecutively enrolled adult patients with MG who were admitted to the Department of Neurology at Ningxia Medical University General Hospital between January 2024 and March 2025 and met the diagnostic criteria for IMC. In this prospective study, participants were assigned to the treatment groups using an alternating enrollment method based on the order of enrollment. Specifically, the first enrolled patient was allocated to the efgartigimod group, the second to the IVIg group, the third to the efgartigimod group, and so forth. Due to the nature of the intervention and the study design, no blinding was implemented.

Inclusion criteria: Clinical diagnosis of MG; Seropositivity for AChR antibodies; Significant worsening of bulbar function (dysarthria, dysphagia) or respiratory muscle function (forced vital capacity [FVC] < 80% predicted) within 2 weeks prior to randomization; Meeting at least one of the following criteria: MGFA type IVb; QMG bulbar subscore ≥ 3;QMG respiratory subscore ≥ 2;Sum of QMG bulbar and respiratory subscores ≥ 4.

Exclusion criteria: Active hepatitis B virus (HBV), hepatitis C virus (HCV), or HIV infection; Treatment with rituximab within 6 months prior to baseline; Administration of IVIg, efgartigimod, or plasma exchange within 4 weeks prior to baseline; Previous treatment with tocilizumab or efgartigimod within 3 months prior to baseline; Baseline immunoglobulin G (IgG) level < 6 g/L; Pregnancy or lactation; Receipt of live or live-attenuated vaccine within 4 weeks prior to baseline; Significant dysfunction of major organs; History of malignancy within the past 3 years (excluding non-melanoma skin cancer);Any other condition considered by investigators to preclude safe participation.

The study protocol was approved by the Institutional Ethics Committee (Approval No. KYLL-2024-1470). Written informed consent was obtained from all participants before any study procedures. Randomization was performed using a computer-generated block randomization sequence. The study was conducted in accordance with the Declaration of Helsinki and Good Clinical Practice guidelines.

### Treatment regimens

Experimental group (efgartigimod): Patients received efgartigimod 10 mg/kg diluted in 100 mL 0.9% sodium chloride solution, administered by intravenous infusion over ≥60 minutes once weekly for 4 consecutive weeks.

Control group (IVIg): Patients received IVIg 400 mg/kg/day administered by intravenous infusion at a rate ≤0.08 mL/kg/min once daily for 5 consecutive days.

All patients maintained their pre-existing background MG therapies, including cholinesterase inhibitors, glucocorticoids, and/or oral immunosuppressants, at stable doses throughout the study period. Concomitant use of other immunomodulatory therapies was prohibited. Identified IMC triggers were managed according to standard clinical practice alongside study treatment.

### Outcome assessment

Assessments were performed at baseline, every 3 days during the first month after treatment initiation, and at months 2 through 6 for patients who achieved IMC remission. All evaluations were conducted by trained assessors using the QMG score and the Myasthenia Gravis Activities of Daily Living (MG-ADL) scale.

Baseline characteristics were retrospectively collected from medical records and included sex, age, disease duration, generalized MG subtype (thymoma-associated, early-onset, or late-onset MG), history of thymectomy, baseline MG treatments, prednisone dosage, MG-ADL score, and MGFA clinical classification.

### Evaluation criteria

Primary efficacy endpoint: Proportion of patients in each group achieving remission from IMC within Month 1 after treatment initiation. Remission from IMC was defined as meeting, for at least 24 hours, any of the following: (i) both QMG bulbar and respiratory subscores < 2; (ii) a combined bulbar plus respiratory QMG score <4; or (iii) improvement to MGFA type < IVb. Time to IMC remission, measured from the first study drug administration to the first time remission criteria were met, was also assessed.

Secondary efficacy endpoints: (i) change from baseline to month 1 in QMG score and subscores (ocular, limb, bulbar, and respiratory); (ii) change from baseline to Month 1 in MG-ADL; (iii) proportion of patients achieving clinically meaningful improvement (CMI), defined as a ≥2-point reduction in MG-ADL score from baseline or a ≥3-point reduction in QMG score from baseline; (iv) proportion of patients achieving Minimal Symptom Expression (MSE), defined as MG-ADL 0 or 1; and (v) subgroup comparisons of QMG respiratory and bulbar subscores among early-onset MG (EOMG), late-onset MG (LOMG), and thymoma-associated MG (TAMG).

### Safety endpoints

Proportion of patients progressing to MC, defined as the need for endotracheal intubation and mechanical ventilation, and the incidence, severity, and relationship to study treatment of all treatment-emergent adverse events (TEAEs).

### Statistical analysis

Data were analyzed using R (version 4.2.1). Continuous variables were assessed for normality (Shapiro–Wilk) and homogeneity of variance (Levene). Variables meeting both assumptions are presented as mean ± standard deviation (SD) and were compared between groups using Student’s t test. Non-normally distributed data are presented as median (interquartile range [IQR]) and were compared using the Mann–Whitney U test (Wilcoxon rank-sum test). Categorical variables are summarized as counts (percentages), with between-group comparisons by Fisher’s exact test. For the primary efficacy endpoint, time to IMC discontinuation was compared between groups using the Wilcoxon rank-sum test, reporting group medians and the between-group median difference with its 95% confidence interval (CI). QMG and MG-ADL scores were analyzed using two-way ANOVA to assess main effects (treatment group, time) and their interaction, with appropriate corrections applied for violations of homogeneity of variance or sphericity. Differences between efgartigimod and IVIg rescue across IMC subgroups were evaluated using a mixed-effects model with subjects as a random effect and fixed effects for treatment, time, subgroup, and their interactions. When model assumptions were not met, parametric inferences were omitted and only descriptive summaries were provided. All tests were two-sided, with P < 0.05 considered statistically significant.

## Results

### Baseline characteristics

A total of 38 patients with AChR–Ab+ IMC were enrolled and randomized to the efgartigimod group (n = 21) or the intravenous IVIg group (n = 17). No statistically significant differences were observed between the groups in demographic characteristics (sex, age), clinical features (disease duration, MGFA type, thymic pathology), or background medications (cholinesterase inhibitors, glucocorticoids, immunosuppressants) (P > 0.05 for all comparisons), indicating balanced baseline characteristics. The efgartigimod group had a male-to-female ratio of 10:11, compared with 8:9 in the IVIg group. The mean age was 58.0 ± 15.4 years in the efgartigimod group and 51.4 ± 15.5 years in the IVIg group. All patients tested positive for AChR antibodies (**Table 1**).

**Table 1:** Patient characteristics, total n = 38.

Variables	Efgartigimod (n=21)	IVIg(n=17)
Age at onset, years, Mean ± SD	58.048 ± 15.445	51.353 ± 15.488
Disease duration, months, M (Q 1, Q 3)	12 (5, 84)	15 (2, 36)
Corticosteroid Dose, mg, M (Q 1, Q 3)	25 (20, 30)	20 (12, 50)
Sex, n (%)		
Female	11 (52.4%)	9 (52.9%)
Male	10 (47.6%)	8 (47.1%)
MGFA class at initial diagnosis, n (%)		
Class IIIb	15 (71.4%)	13 (76.5%)
Class IVb	6 (28.6%)	4 (23.5%)
Thymus histology, n (%)		
Hyperplasic thymus	1 (4.8%)	0 (0%)
Thymoma	7 (33.3%)	4 (23.5%)
History of Thymus Surgery, n (%)	7 (33.3%)	4 (23.5%)
Treatment at baseline, n (%)		
Tacrolimus	16 (76.2%)	13 (76.5%)
Mycophenolate mofetil	4 (19%)	4 (23.5%)
Azathioprine	1 (4.8%)	0 (0%)
Pyridostigmine at enrollment, n (%)	20 (95.2%)	17 (100%)
Corticosteroid at enrollment, n (%)	17 (81%)	16 (94.1%)
IMC trigger, n (%)		
None	5 (13.2%)	5 (13.2%)
Non-adherence to medication	3 (7.9%)	1 (2.6%)
Infection	3 (7.9%)	5 (13.2%)
Overexertion	4 (10.5%)	5 (13.2%)
Post-thymectomy	6 (15.8%)	1 (2.6%)

### Efficacy of efgartigimod in IMC management

**Figure 1:**
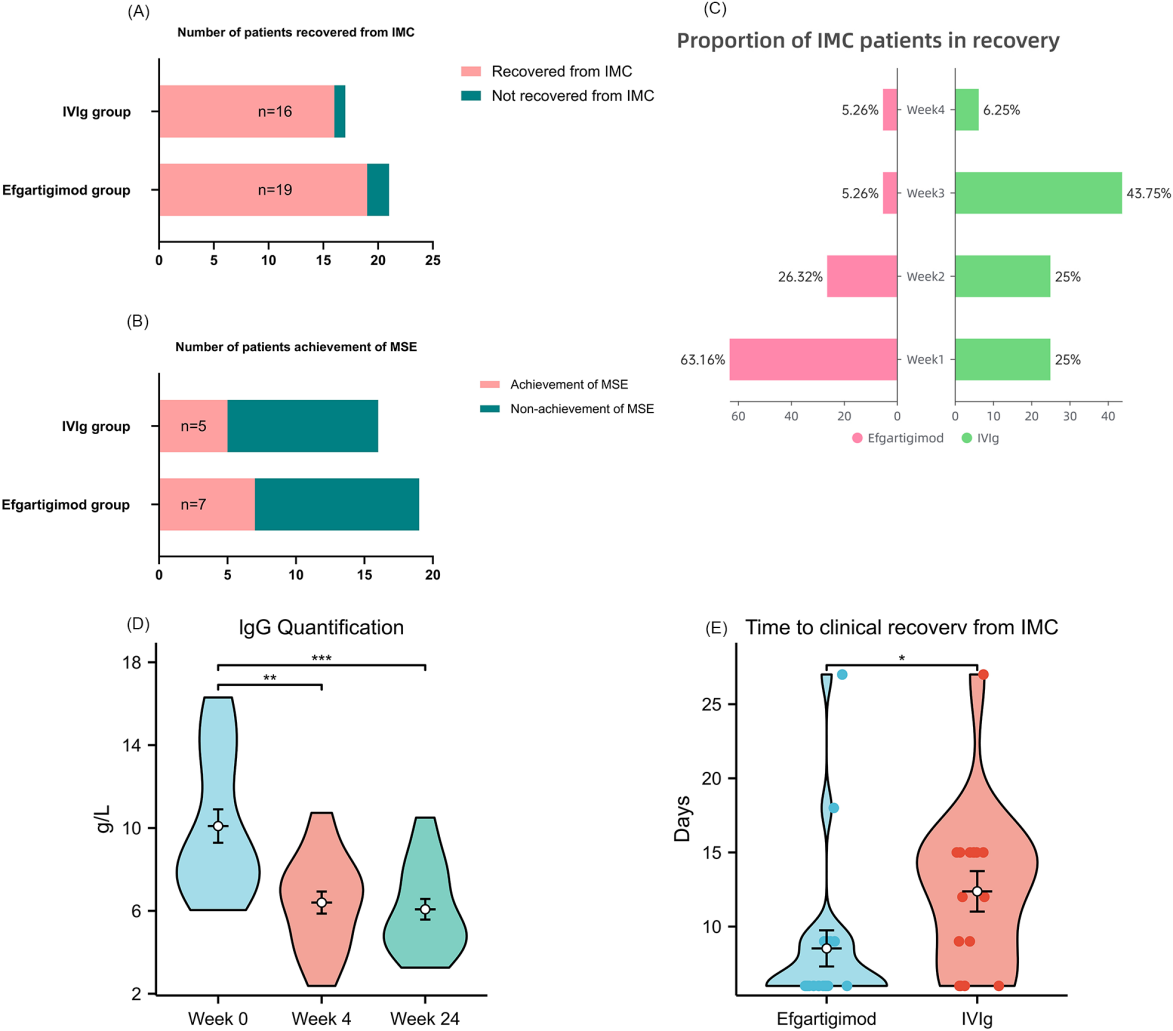
Compares multiple outcomes in patients with IMC treated with efgartigimod versus IVIg. **(A, C, D)** display the primary outcomes. **(A)** shows the number of patients recovered from IMC; **(C)** shows the proportion of IMC patients in recovery; **(D)** shows the time to clinical recovery from IMC. Remission from IMC was defined as meeting, for at least 24 hours, any of the following: (i) both QMG bulbar and respiratory subscores < 2; (ii) a combined bulbar plus respiratory QMG score <4; or (iii) improvement to MGFA type <IVb. **(B)** shows the number of patients achieving MSE (ADL ≤ 1) at 4 weeks after treatment. **(E)** shows IgG quantification at baseline, 4 weeks after treatment, and 24 weeks after treatment.

Within 1 month of treatment initiation, the proportion of patients who achieved clinical recovery from IMC did not differ significantly between the efgartigimod and IVIg groups (19/21 [90.48%] vs 16/17 [94.12%]; RR = 0.96; 95% P = 1.00, **Figure 1A**).Significantly more patients achieved remission within the first week with efgartigimod than with IVIg (63.16% vs. 25%; P<0.05). In contrast, the majority of IVIg-treated patients (5.26% vs 43.75%;P<0.01) achieved remission during the third week (**Figure 1C**).The mean time to clinical recovery from IMC was significantly shorter with efgartigimod than with IVIg (8.53 ± 3.21 days vs 12.38 ± 4.56 days; P = 0.011; **Figure 1D**).

### QMG total score and subscores

**Figure 2:**
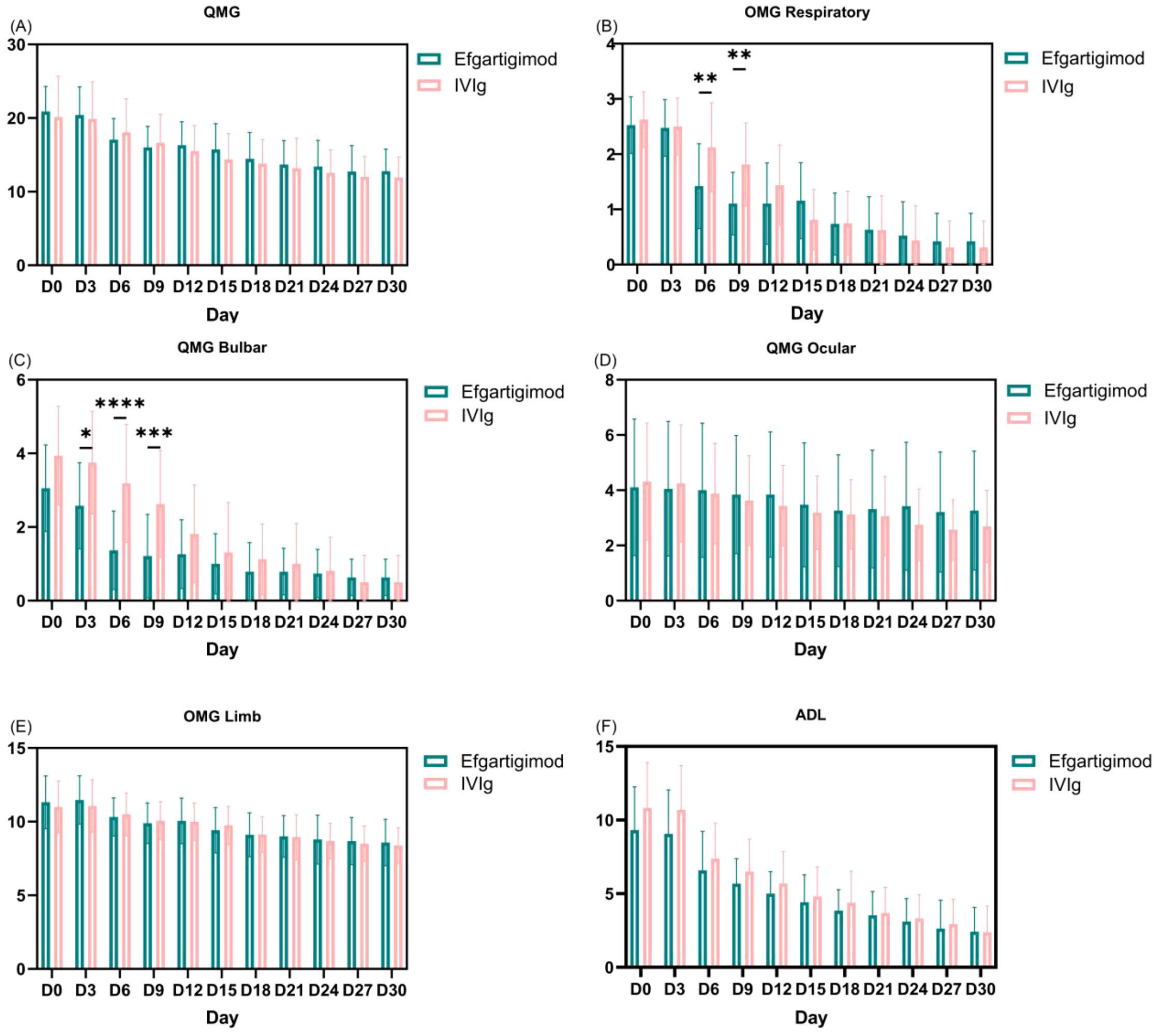
**(A-E)** display Change from baseline to month 1 in QMG score and subscores. QMG subscores included diplopia, ptosis, eye closure, respiratory (vital capacity), axial muscle (head-lifted), limb (right and left arms outstretched, hand grip strength, right and left legs outstretched), and bulbar (swallowing and speech). The respiratory subscore ranges from 0 to 3.The bulbar subscore ranges from 0 to 6.The ocular subscore ranges from 0 to 6. The limb subscore ranges from 0 to 18. Panel **(F)** change from baseline to Month 1 in MG-ADL.

Analyses of changes from baseline demonstrated clinically meaningful improvements with both efgartigimod and IVIg over 30 days. The mean total QMG score decreased from 20.3 ± 4.2 at baseline to 12.1 ± 3.8 on Day 30, corresponding to a reduction of 8.2 points (approximately 40% of baseline). Between-group differences in the change from baseline were not statistically significant (P>0.05; **Figure 2A**). Lower QMG scores indicate improvement. All QMG subscores improved from baseline across muscle groups (**Figures 2B–E**), with the most pronounced gains observed in the respiratory and bulbar domains. In the efgartigimod group, respiratory subscores improved earlier, with mean changes of 1.11 points on Day 6 and 1.81 points on Day 9 (P < 0.01; **Figure 2B**). In contrast, the IVIg group showed a significant respiratory improvement at Day 15 (−1.47 points; P < 0.01; **Figure 2B**).For bulbar function, the efgartigimod group demonstrated significant improvement as early as Day 6 (−1.68 points; P < 0.01), whereas the IVIg group reached statistical significance at Day 12 (−2.13 points; P < 0.01; **Figure 2C**).

### Changes in MG-ADL scores

At baseline, mean MG-ADL total scores were 9.32 in the efgartigimod group and 10.81 in the IVIg group, reflecting similar levels of functional impairment (lower scores indicate less impairment). At Day 30, MG-ADL total scores decreased to 6.9 and 8.4 in the efgartigimod and IVIg groups, respectively (**Figure 2F**), corresponding to mean reductions of 2.42 and 2.41 points from baseline. These decreases are consistent with improvements in activities of daily living. For the secondary endpoint, minimal symptom expression (MSE) at Day 30 was achieved by 7 of 19 patients (36.84%) in the efgartigimod group and 5 of 16 (31.25%) in the IVIg group. There was no statistically significant between-group difference (p>0.05) (**Figure 1B**), indicating similar short-term rates of achieving MSE in this cohort.

### Subtypes analysis of efgartigimod versus IVIg in IMC

**Figure 3:**
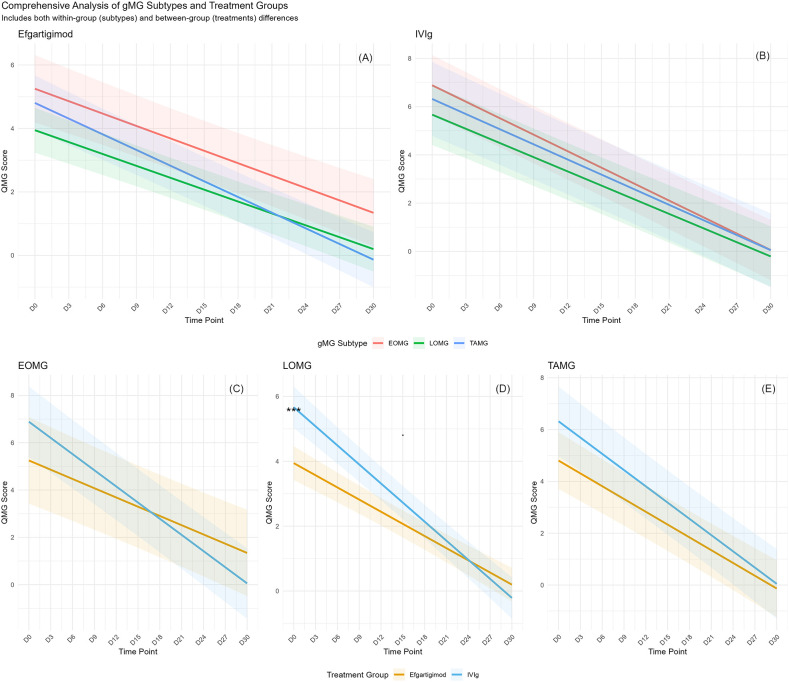
Comprehensive analysis of gMG subtypes and treatment groups included both within-group (subtypes) and between-group (treatments) differences. **(A, B)** Subtypes Analysis of Efgartigimod versus IVIg in IMC. **(C–E)** subgroup comparisons of QMG respiratory and bulbar subscores among EOMG,LOMG, and TAMG. The respiratory and bulbar subscore ranges from 0 to 9.

**Figure 4:**
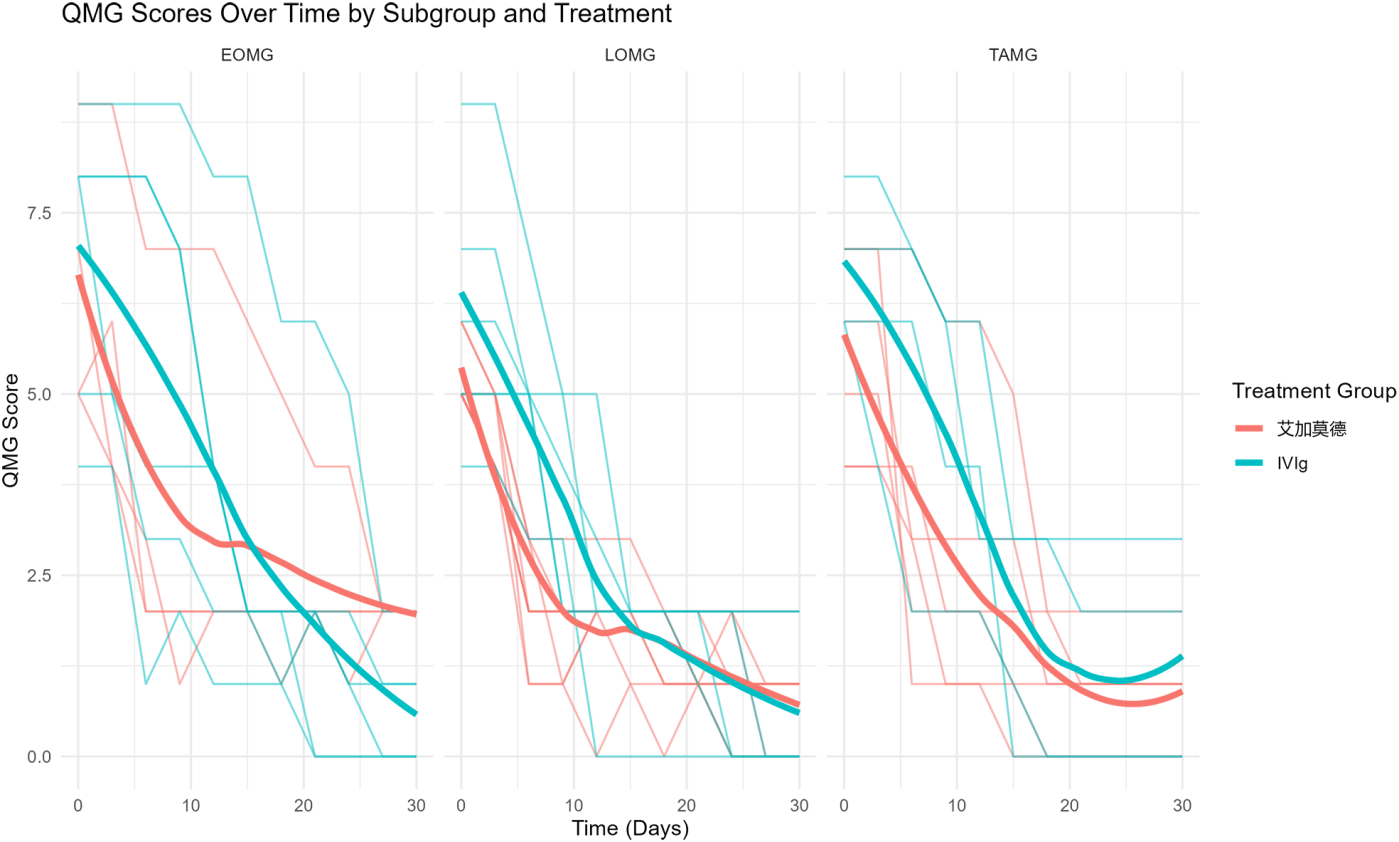
Subgroup comparisons of QMG respiratory and bulbar subscores among EOMG, LOMG, and TAMG.

Among the 38 patients with AChR-Ab+ IMC included in this study, the baseline distribution of EOMG, LOMG, and TAMG subtypes was as follows: 10 patients had EOMG (4 in the efgartigimod group and 6 in the IVIg group), 15 had LOMG (9 in the efgartigimod group and 6 in the IVIg group), and 10 had TAMG (6 in the efgartigimod group and 4 in the IVIg group).Both efgartigimod and IVIg were associated with significant reductions in QMG scores from baseline across the EOMG, LOMG, and TAMG subgroups (p < 0.05, **Figures 3A, B**). Lower QMG scores indicate clinical improvement. Between-group comparisons using mixed-effects models adjusted for baseline evaluated interval-specific trajectories of change. During Days 0–15, efgartigimod showed greater numerical improvement than IVIg in EOMG (1.31 ± 0.65 vs 1.22 ± 0.90; p = 0.128) and LOMG (−0.86 ± 0.57 vs −0.65 ± 1.00; p = 0.302); these differences were not statistically significant (**Figures 3C, D**, **4**).In TAMG, efgartigimod demonstrated numerically greater improvement during Days 15–30 compared with IVIg (1.48 ± 0.70 vs 0.01 ± 1.00, **Figures 3E**, **4**). Post–Day 15, the rate of QMG reduction was faster in the IVIg group within EOMG (**Figures 3C**, **4**). In LOMG, improvement rates after Day 15 were similar between treatments (**Figures 3D**, **4**).Not all between-group comparisons reached statistical significance at the prespecified.

### Disease recurrence within 6 months after successful rescue

Following resolution of the acute crisis and weaning from intensive care, maintenance regimens differed between groups. Most patients initially treated with efgartigimod (15/19) continued on-demand efgartigimod infusions alongside baseline oral therapy (e.g., cholinesterase inhibitors and/or immunosuppressants), whereas all patients in the IVIg group received only baseline oral medications. For subgroup analysis, patients who had been weaned from intensive care were categorized as follows: Group A: Initial efgartigimod therapy + no maintenance therapy (n=4),Group B: Initial efgartigimod therapy + on-demand efgartigimod maintenance (n=15),Group C: Initial IVIg therapy + no maintenance therapy (n=16).The primary endpoint was the clinical relapse rate within 6 months after leaving intensive care, defined as requiring readmission or a recurrence of a QMG respiratory/bulbar subscore ≥4.Relapse occurred in 0% (0/4) of Group A and 6.7% (1/15) of Group B, both numerically lower than the 31.3% (5/16) observed in Group C. Pairwise comparisons did not reach statistical significance (Group A vs. Group C, p=0.28; Group B vs. Group C, p=0.073).During the follow-up period, the proportion of patients hospitalized for disease exacerbation was highest in Group C (31.25%), whereas no exacerbation-related hospitalizations occurred in Group A. With respect to exacerbation triggers, infection was the sole identified trigger in Group B (100%), while triggers in Group C were more heterogeneous, with overexertion being the most frequently reported (40%). Overall, Group B differed from the other groups in terms of disease duration, age at onset, and medication use, whereas Group C appeared to have a higher risk of disease exacerbation. After 6 months of follow-up, corticosteroid doses decreased overall. Group B maintained the lowest dose (17.69 mg/day), representing a reduction of nearly 12 mg/day from baseline, whereas Group C had the highest dose (27.32 mg/day), with a reduction of 6.32 mg/day from baseline. (**Supplementary Table 1**) Serum IgG levels: Among the 19 patients in the efgartigimod cohort who were weaned from intensive care, serum total IgG was measured at baseline, 1 month, and 6 months after treatment initiation. The baseline total IgG level was 8.43 g/L; after 1 month, it decreased significantly to 6.51 g/L (P < 0.01). In the 15 patients who continued maintenance efgartigimod, the total IgG at 6 months was 5.37 g/L, with no significant further decrease relative to the 1 month level (**Figure 1E**).

### Safety

One patient in each group experienced myasthenic crisis (efgartigimod: 1/21 [4.76%]; IVIg: 1/17 [5.88%]), with no significant between-group difference (p=1.00). No serious adverse events (SAEs) were reported, and no treatment-emergent adverse events (TEAEs) led to permanent treatment discontinuation in either group during the treatment period.

## Discussion

This prospective comparative cohort study used alternating allocation, which may have partially reduced selection bias. Conducted under real-world clinical conditions, the findings may offer reference for clinical decision-making. The study focused on AChR-Ab–positive IMC and distinguished clinical subtypes, which may help inform individualized treatment strategies (16). Although IMC resolution rates did not differ significantly between groups, the mean time to IMC resolution was shorter with efgartigimod than with IVIg (8.53 vs 12.38 days; P = 0.011). By the first week of treatment, the efgartigimod group showed a 32.1% reduction from baseline in QMG bulbar and respiratory subscores (P < 0.01), whereas comparable improvement in the IVIg group was observed at week 2. These temporal differences in response appear consistent with the known pharmacologic profiles of the two agents. As an FcRn antagonist, efgartigimod reduces serum IgG levels by approximately 65%–75% within 24–48 hours after administration, thereby accelerating clearance of pathogenic antibodies. This mechanism has been reported in the international multicenter Phase III ADAPT trial and in Zhang et al.’s retrospective analysis of a Chinese cohort. This mechanism has been corroborated by the international multicenter Phase III ADAPT trial and by Zhang et al.’s retrospective analysis of a Chinese cohort (11, 17). In contrast, IVIg exerts progressive immunomodulatory effects through multiple pathways, including Fcγ receptor–mediated suppression, induction of anti-inflammatory mediators, and complement neutralization, resulting in a relatively delayed clinical onset consistent with international guideline descriptions (18–20).Despite these differences in onset, one-month IMC resolution rates were broadly comparable between groups (90.48% vs 94.12%), suggesting that both therapies can serve as rescue options, albeit potentially in different clinical contexts. For rapidly progressive IMC—such as post−infectious acute exacerbations—where prompt reduction of antibody burden is considered important to control disease progression, efgartigimod may offer an advantage in early antibody clearance and could potentially shorten the duration of mechanical ventilation and lower the risk of related complications. Conversely, IVIg remains a relevant option for IMC with slower disease progression, particularly when broader immunomodulatory effects are desired. By the end of treatment, improvements in total QMG scores were generally similar between groups, which is consistent with comparable overall effects on myasthenia gravis severity. Subgroup analyses indicated that respiratory and bulbar muscle function appeared to improve earlier with efgartigimod—by approximately 6 and 9 days, respectively—than with IVIg. These observations are in line with the critical care emphasis on early intervention and rapid stabilization reported in recent studies, and may be particularly relevant for patients at higher risk due to bulbar and/or respiratory involvement (21).

At day 30, MG-ADL scores improved significantly in both treatment arms, indicating better activities of daily living. However, the proportion of patients achieving minimal symptom expression (MSE) at day 30 did not differ significantly between arms, further supporting the comparable efficacy of the two strategies with respect to functional endpoints. These findings align with international consensus guidance endorsing multimodal, synergistic therapy for myasthenia gravis and support individualized treatment selection based on patient characteristics, disease severity and acuity, and drug availability (22).

For long-term disease management, 6−month relapse rates differed among IMC patients who achieved initial treatment success. Relapse occurred in 0% (0/4) in the efgartigimod group without maintenance therapy (Group A) and 6.7% (1/15) in the on−demand maintenance group (Group B), both numerically lower than in the IVIg group without maintenance therapy (31.3%, 5/16; Group C). The absence of relapses in Group A should be interpreted with caution because of the very small sample size (n = 4), which limits the precision and robustness of this estimate. In addition, potential baseline differences among groups—such as heterogeneity in disease characteristics or concomitant treatments—cannot be excluded and may have influenced the observed relapse patterns. The single relapse in Group B also suggests that an on−demand maintenance approach requires close follow−up to detect and manage early signs of recurrence. Across all patients initially treated with efgartigimod, the overall 6−month relapse rate was 5.3% (1/19), which was numerically lower than that observed in the IVIg cohort (31.3%, 5/16). This difference should be interpreted in the context of the limited sample size and non-randomized design, and requires confirmation in larger, controlled studies (17, 23). In contrast, IVIg exerts broad, non-specific immunomodulatory effects with a relatively short duration of action, making sustained suppression of pathogenic antibodies more challenging and potentially contributing to higher relapse rates—consistent with real-world data from China (24, 25). The relapse rate observed in Group B is in line with long-term outcomes reported for FcRn antagonists, supporting the role of maintenance therapy in patients with chronic active disease.

The overall safety profiles were comparable between the two groups, with similar rates of progression to myasthenic crisis (efgartigimod: 1/21 [4.8%]; IVIg: 1/17 [5.9%]). However, the treatment failure cases highlight critical considerations for individualized risk management. Overall safety profiles were comparable between groups: progression to myasthenic crisis occurred in 1/21 (4.8%) patients treated with efgartigimod and 1/17 (5.9%) patients treated with IVIg. Given the small numbers, these data are descriptive and do not support formal between-group comparison. Nevertheless, the treatment-failure cases highlight important considerations for individualized risk management. In the efgartigimod arm, two patients who progressed had notable comorbid contexts—one with active pulmonary infection and one with refractory MG after thymectomy. These case-level observations suggest that IgG-lowering alone may be insufficient to mitigate infection-driven inflammation or thymoma-associated, potentially T−cell–mediated, autoimmunity. In the IVIg arm, the single progression occurred in a patient with concomitant Guillain–Barré syndrome and an unfavorable clinical course, underscoring that broad immunomodulation may be inadequate in complex autoimmune overlap disorders. These observations are hypothesis-generating and point to the potential value of stratified management strategies for myasthenic crisis: infection-triggered cases may require pairing antibody-lowering interventions with timely antimicrobial therapy and source control (26); thymoma-associated cases may warrant evaluation of FcRn antagonists in combination with complement inhibition; and patients with overlapping autoimmune neurologic diseases benefit from multidisciplinary care planning (26). Moreover, because FcRn antagonism accelerates IgG catabolism, vigilance for infections is warranted—particularly in patients with borderline low baseline IgG—through clinical surveillance and periodic IgG monitoring. No treatment-emergent adverse events (TEAEs) were recorded in either group, which may be attributable to the small sample size. Per the existing literature, efgartigimod’s commonly reported adverse events include headache (15–20%), upper respiratory tract infection (10–15%), and mild hypogammaglobulinemia (5–10%), which typically do not require treatment interruption (27).In the IVIg group, although we administered routine prophylactic low−molecular−weight heparin calcium (4,000 IU daily) and observed no thromboembolic events, recognized risks of IVIg remain, including acute kidney injury (approximately 5–8%), hemolytic reactions (3–5%), and allergic reactions (1–3%) (28).

This study has several limitations: The small sample size (n = 38) renders the study underpowered for subgroup analyses of treatment effect by antibody subtype (AChR vs. MuSK) and by the presence or absence of thymoma. The limitations in randomization lie in the use of alternating allocation rather than strict randomization, and the absence of blinding may have introduced detection and performance bias. The single-center design may limit the external validity and generalizability of the findings. The 6-month follow-up is insufficient to assess durable efficacy and late safety signals. The open-label design is subject to performance and detection bias. Future research should focus on, Develop predictive biomarkers, such as serial (longitudinal) antibody titer kinetics or polymorphisms in the neonatal Fc receptor gene (FCGRT).Evaluate combination therapy strategies, including FcRn antagonists paired with complement inhibitors or B-cell–depleting therapies. Extend investigations to pediatric populations and patients with complex comorbidities.

## Conclusion

Efgartigimod demonstrated comparable efficacy to IVIg for IMC remission but with a significantly faster onset, supporting its role as a rapid and safe alternative.

## Data Availability

The raw data supporting the conclusions of this article will be made available by the authors, without undue reservation.
