# Supplementary material for: Efficacy and safety of efgartigimod in the treatment of impending myasthenic crisis
Source: Front Immunol. 2026 Mar 12;17:1760953. doi: 10.3389/fimmu.2026.1760953 (PMC13017867; doi:10.3389/fimmu.2026.1760953)
Supplement: Supplementary file 1 [file Table1.docx]

| Variables | Group A (n=4) | Group B (n=15) | Group C (n=16) |
| --- | --- | --- | --- |
| Sex, n (%) |  |  |  |
| Female | 1 (25%) | 9 (60%) | 9 (56.25%) |
| Male | 3 (75%) | 6 (40%) | 7 (43.75%) |
| Age at onset, years | 53.25 ± 12.066 | 58.933 ± 17.148 | 50.812 ± 15.829 |
| Disease duration, months | 18 (12, 42) | 24 (2.5, 90) | 16.5 (2.75, 42) |
| MGFA class at initial diagnosis,  n (%) |  |  |  |
| Class Ⅲb | 1 (25%) | 4 (26.67%) | 4 (25%) |
| ClassⅣb | 3 (75%) | 11 (73.33%) | 12 (75%) |
| Corticosteroids Dose after detachment from IMC,mg | 33.75 | 29.61 | 33.45 |
| Immunosuppressant therapy  after 6 months of follow-up n (%) |  |  |  |
| Tacrolimus | 2 (50%) | 13 (86.67%) | 12 (75%) |
| Mycophenolate mofetil | 1 (25%) | 2 (13.33%) | 4 (25%) |
| Azathioprine | 1 (25%) | 0 (0%) | 0 (0%) |
| Corticosteroids Dose after 6 months of follow-up,mg | 25 | 17.69 | 27.32 |
| Aggravated illness hospitalized, n (%) | 0 (0%) | 1 (6.67%) | 5 (31.25%) |
| Aggravation trigger, n (%) |  |  |  |
| None | 0 | 0 | 1 (20%) |
| Non-adherence to medication | 0 | 0 | 1 (20%) |
| Infection | 0 | 1(100%) | 1 (20%) |
| Overexertion | 0 | 0 | 2 (40%) |

Supplementary Table 1: Baseline characteristics of patients with 6‑month follow‑up.
